# Temperature-Based Prediction of Joint Hardness in TIG Welding of Inconel 600, 625 and 718 Nickel Superalloys

**DOI:** 10.3390/ma14020442

**Published:** 2021-01-18

**Authors:** Wojciech Jamrozik, Jacek Górka, Tomasz Kik

**Affiliations:** 1 Department of Fundamentals of Machinery Design, Silesian University of Technology, Konarskiego Str. 18a, 44-100 Gliwice, Poland; 2 Department of Welding Engineering, Silesian University of Technology, Konarskiego Str. 18a, 44-100 Gliwice, Poland; jacek.gorka@polsl.pl (J.G.); tomasz.kik@polsl.pl (T.K.)

**Keywords:** TIG welding, Inconel superalloys, thermography, hardness, prediction

## Abstract

Welding is an important process in terms of manufacturing components for various types of machines and structures. One of the vital and still unsolved issues is determining the quality and properties welded joint in an online manner. In this paper, a technique for prediction of joint hardness based on the sequence of thermogram acquired during welding process is proposed. First, the correspondence between temperature, welding linear energy and hardness was revealed and confirmed using correlation analysis. Using a linear regression model, relations between temperature and hardness were described. According to obtained results in the joint area, prediction error was as low as 1.25%, while for HAZ it exceeded 15%. Future work on optimizing model and input data for HAZ hardness prediction are planned.

## 1. Introduction

The development of many industries important for the national economy, especially the energy, chemical, food, aviation and space industries, as well as marine and nuclear engineering and environmental protection, requires the development of new technologies for the production of metallic materials and their products using both high mechanical properties and resistance to highly aggressive corrosive environments, in particular, gas corrosion at elevated operating temperatures [1,2]. Materials that meet specific technological and operational requirements, especially in terms of heat resistance and creep resistance, as well as creep resistance at high temperatures (even up to 1100 °C) and resistance to aggressive working environments while maintaining a low thermal expansion coefficient and relatively good weldability, include, next to cobalt and titanium alloys, nickel superalloys of the Inconel type [3,4,5]. Thin sheets and pipes welded components and assemblies can be found in aircraft engine bleed air applications, fuel systems and other aircraft ECS (environmental control systems), potable water tanks, fuel tanks, hydraulic reservoirs, heat exchangers, boilers, exhaust systems of motorcycles, cars and aircrafts [6,7,8,9].

Inconel nickel-chromium superalloys contain approximately 15–20% chromium and iron additives up to approximately 18%, molybdenum up to approximately 16%, niobium up to approximately 5% and other elements (Co, Cu, W). They are characterized by high corrosion resistance and high durability at temperatures up to approximately 1000 °C. They are used in the most thermally loaded parts of jet engines, accounting for nearly 50% of their mass [10].

Research on nickel and its alloys is currently widely continued [11,12,13,14,15,16,17,18,19,20,21]. They concern, inter alia, bonding technologies, such as soldering [14], welding of construction elements operated in high temperature and aggressive environment [15], welding Ni alloys with non-alloy steels and structural analysis of welded joints [16], as well as joining bimetal with a clad Ni alloys [17], or laser welding of Inconel 625 superalloy fin tubes [18] and the formation of surface layers by thermal spraying and welding [19,20]. However, it seems that the research issues of welded joints of thin sheets of Inconel alloys are presented in the available literature only as a contribution.

Assessing the joint quality and the stability of welding process for automatic control and evaluation of welded joints is assessed using a large variety of sensing techniques. Vision sensing, including passive vision and active vision, has been widely utilized in welding process monitoring in different wavelength of imaging devices (visible, infrared, ultraviolet). A special passive vision system for seam tracking in robotic GTAW and GMAW has been designed and successfully applied [21]. A 3D active vision system was elaborated [22,23,24]. This system allows the automated predictive control of weld penetration. Novel methods for feature extraction from 3D vision systems are presented in [25,26,27]. Vision systems are often applied; there must be a good access to the welding pool and solidified seam area, to obtain images of proper quality. Additionally, active vision systems are often complex solutions because an external excitation is demanded in their case. Sensitivity and applicability of the welding acoustic signal was also studied [28], and acoustic sensing has a growing interest of many research teams (ultrasound and acoustic bands) [29,30,31]. There were special features extracted from the acoustic pressure signals. Analyses in frequency domain and time–frequency transformations were used, to assess the weld process stability. The physical significance of the features was found. The arc sound sensing system is in most cases simpler than the vision sensing system; however, it needs accurate calibration [32], and noise from welding machine cannot be ignored, but it can be identified and, in most cases, filtered out without removing significant informational content from the acoustic signal. Other group of monitoring methods utilizes electric signals of welding process that are, in most cases, easy to collect (especially for GMAW process), including the signals of arc current [33] and arc voltage [34]. The arc electrical signal is easy to obtain, and the sensing system does not limit the weld accessibility. Moreover, thermal imagining and multisensory fused data can be used to assess weld state [35,36]. For the TIG method [37,38,39,40,41], measurement of electric parameters with high frequency demands a dedicated device, that is protected against high voltage, that is generated at the stage of arc ignition. This high voltage, that is, at the level of several thousand volts, is generated by an ionizer that allows arc ignition without contact of the electrode and welded workpiece. In this case, monitoring and assessing the welding process and produced joint with an imagining device, that is, working in the visible light range or in the infrared, is a better option. Additionally, using an IR camera allows the measurement and differentiation of measurement results for characteristic zones, that are present on a joint cross-section, like HAZ (heat-affected zone), fusion zone, welded seam and base material [42,43]. According to that transformation, that occurs in all those zones can be identified and controlled in term of properties obtained. All of the research studies mentioned above are focused mainly on process control and stability assessment. There is little attention paid on the referencing of weld mechanical properties while the weld is produced. In this paper, the influence of welding process performed with different parameters is investigated in order to reveal a linear model that describes the relationship between temperature measured on the weld surface and hardness in the HAZ and weld area.

In this paper, research on the possibility of assessing and predicting the hardness in joint and in HAZ based on temperature distribution acquired by an IR camera is presented. Metal plates made form nickel superalloys type Inconel 600, 625 and 718 were joined using the TIG method, and temperature distribution on the surface of plates was recorded. After welding for all samples, the HV1 hardness was measured on the joint cross section. Correlating obtained measurement results, a relationship between temperature, welding linear energy and hardness was confirmed. A linear regression model was elaborated and quantified to model and predict hardness in seam and HAZ of welded joint.

## 2. Materials and Methods

The tests were carried out on the joints of thin sheets with a thickness of 1 mm of nickel superalloys type Inconel 600, 625 and 718 welded by TIG method. The sheets used to make the welds came from the industrial process of Huntington Alloys Corporation (Huntington, WV, USA) involving the smelting of Inconel 600 superalloy ingots in an electric furnace, while Inconel 625 and 718 were treated in a vacuum furnace. Next, a plastic processing by cold rolling with intermediate heat treatment (recrystallization annealing) was performed. The chemical composition of the tested sheets is shown in Table 1.

**Figure 1:**
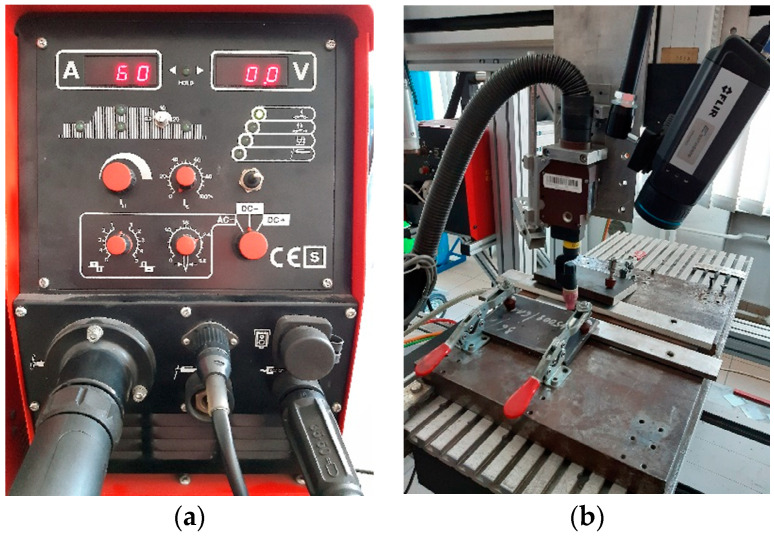
Welding device (**a**) and TIG torch (**b**) used during studies.

The Casto TIG 2002 device (Castolin GmbH, Kriftel, Germany) was used for welding sheets from the investigated Inconel nickel superalloys (Figure 1). TIG welding of sheets was carried out in laboratory conditions with the following constant parameters: welding speed 3 mm/s, shielding gas Ar 12 L/min, ridge shielding gas Ar 3 L/min, tungsten electrode (thoriated), WT20 with a diameter of 2.4 mm.

Measurement of temperature distribution in welded joints was made with FILR A655sc infrared camera (FLIR Systems, Inc., Wilsonville, OR, USA) (Figure 1). The spatial resolution of camera was 640 × 480 px, and the emissivity was set on a constant level ε = 0.4. The emissivity value was estimated for temperature of 600 °C, when sample covered partially with a graphite-based spray with high emissivity was heated in an oven. Emissivity estimated in that trial was ε = 0.2. Taking into consideration the fact that for higher temperatures, the emissivity will increase in longer wavelength, it was assumed that the emissivity taken for measurement will be ε = 0.4 for all materials. Variations of emissivity in function of temperature were not taken into consideration because in the proposed method, there is no need to compare temperatures between molten and solidified regions of joint. IR images were taken with 60 fps. The optical axis of camera was inclined to the plane of the sample at an angle of 30 degrees and the distance between camera and welded sample was 300 mm. On thermograms, the welding pool as well as the area of solidified and cooling joint was acquired.

The welding device was operating in a constant current mode, thus the arc voltage was slightly changing during the process, to compensate momentary instabilities of the process. In Table 2, welding parameters that were changed during the experiment are gathered. Additionally, linear energy of welding was calculated using the following equation:

(1) $$E=k\frac{UI}{v}\;[J/\mathrm{mm}]$$

where: *k* is the process thermal efficiency coefficient (0.6 for the TIG process), *U* is the welding current (A), *I* is the arc voltage (V), and *v* is the welding speed.

**Figure 2:**
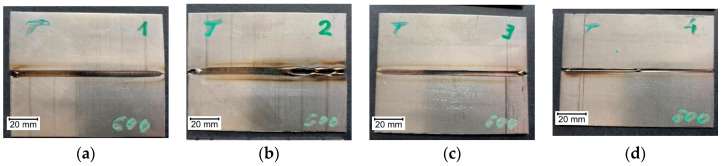
Weld faces made on Inconel 600 sheets, with current: (**a**) A1—35A, (**b**) A2—40A, (**c**) A3—30A, (**d**) A4—25A.

**Figure 3:**
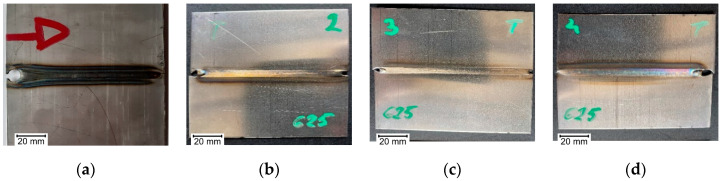
Weld faces made on Inconel 625 sheets, with current: (**a**) B1—50A, (**b**) B2—40A, (**c**) B3—35A, (**d**) B4—45A.

**Figure 4:**
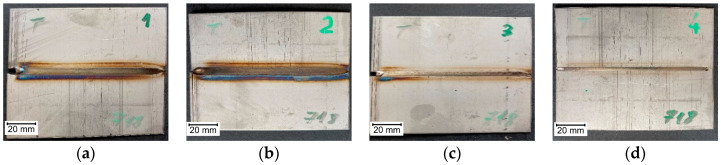
Weld faces made on Inconel 718 sheets, with current: (**a**) C1—35A, (**b**) C2—40A, (**c**) C3—30A, (**d**) C4—25A.

Views of examined sample joints are presented in Figure 2, Figure 3 and Figure 4. It can be seen that in sample A2 (Figure 2b), there are burnouts connected with the high heat that was delivered to the joint. For specimens that were welded with minimal current, the weld face is narrow, and joints are characterized with a lack of fusion. All welding inconsistencies were confirmed by digital X-ray examination. Because of meal sheet thickness, there were no inclusions or porosity detected in made joints. For the tested joints, burnout (510) occurred for the extreme (high) value of the current in the case of Inconel 600 sheets. There is also a lack of connection due to the linear misalignment (507) for the A4 sample (Inconel 600). Additionally, there are also temper colors (610).

Macroscopic metallographic tests were carried out on samples of joints cut perpendicular to the weld line of the welded sheets. The cut samples were mechanically ground on abrasive papers of various granulation of the abrasive, successively from 100 to 800, and polished on polishing wheels cooled with water. In order to reveal the joint zone (S) and the heat-affected zone (HAZ) and the area of the base material (BR), etching of the joint surfaces with Adler’s reagent was carried out with the following chemical composition:

- 3 g (NH_4_) 3 (CuCl_4_)
- 20 mL distilled water,
- 50 mL hydrochloric acid HCl,
- 15 g iron chloride FeCl_3_.

The etching of the specimens was carried out in stages during 10 to 15 s at room temperature. Macroscopic observations of the etched joint samples were carried out using an OLYMPUS GX71 light microscope (Olympus Corporation, Tokyo, Japan) with a magnification of up to 50×.

**Figure 5:**
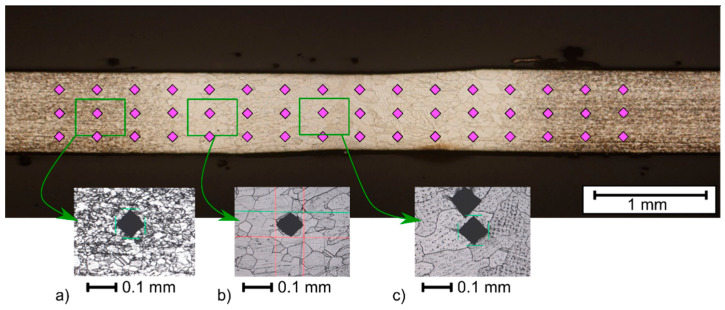
Markers for points used for the hardness measurement at first stage of evaluation. Hardness of chosen exemplary points at sample A3: (**a**) 187HV1; (**b**) 151HV1; (**c**) 160HV1.

**Figure 6:**
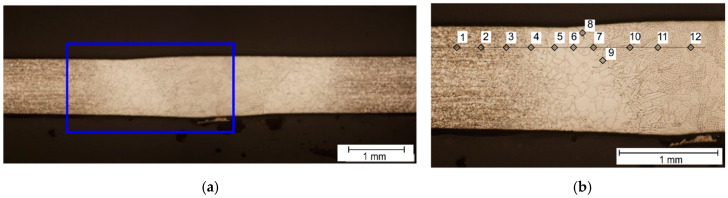
Markers for points used for the hardness measurement according to the ISO 9015-1:2001. (**a**) location of measurement area; (**b**) locations of measurement points.

The hardness tests of welded joints of Inconel nickel superalloys were carried out using the Vickers method using a Struers Duramin 500 (Struers, Ballerup, Denmark) and Hauser hardness tester (Henri Hauser AG, Biel, Switzerland) and using a load of 10 N and a measurement time of 15 s. The measurement was carried out by pressing each time a diamond indenter in the form of a pyramid with a square base and a wall opening angle of 136° in the tested material. In the tested area of the weld, the heat-affected zone and the base material, three hardness measurements were made. The diagram of hardness measurements of the tested joints is shown in the macrophotography (Figure 5 and Figure 6). Hardness measurements were made at samples cross sections localized in the middle of each specimen, beside samples A2 and A4, where sections were made in the middle of stable weld (according to the presence of burnouts).

## 3. Results and Discussion

### 3.1. Hardness Measurement Results

At the first stage of joint hardness evaluation, the Vickers hardness for 1 kg load was measured. To check the spread of measurement, and to verify that the hardness is mainly affected by the grain size, a dense measurement pattern was applied (Figure 5). In the *x*-axis, idents were separated by 0.3 mm, while in the *y*-axis, the offset was 0.2 mm. The measurement was performed for one chosen sample for each material. It was found that for all materials, the standard deviation is less than 3% of the mean value in the base material, HAZ and the joint zone. After that, it was decided to perform measurements according to ISO 9015-1:2001 standard: Destructive tests on welds in metallic materials—Hardness testing—Part 1: Hardness test on arc welded joints. It will make possible to apply proposed approach, having results from commonly made measurement procedure. In the case of limited number of measuring points, main zones of welded sheet joints from the weld area (seam, S) through the HAZ to the base material were considered. Moreover, two the transient zones marked as TZ1 (BM/HAZ) and TZ2 (S/HAZ) were included. In Figure 6, there are points of hardness measurement marked, and hardness values are presented in Table 3.

**Figure 7:**
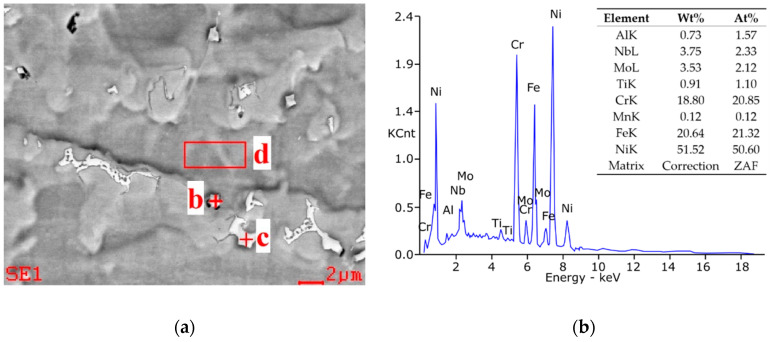
High-resolution transmission electron microscopy (HRTEM) image of weld area in Inconel 718 sample: (**a**) localization of measurement spots at the sample dendritic structure, (**b**) spectrogram of the interdendritic precipitations components (spot C) with a table of concentrations of the analyzed elements, made by energy-dispersive X-ray spectroscopy (EDS).

The hardness of the base material of the tested nickel superalloys is diversified and is on average about 186 HV for Inconel 600 superalloy and about 253 ÷ 256 HV in the case of the Inconel 625 and 718 superalloys. The higher BM hardness of the Inconel 625 and 718 superalloys results from the presence of a greater amount of carbide-forming elements in these superalloys, such as Cr, Mo as well as Nb and Ti, which determine their structure and complex phase composition and greater strength in solution and precipitation in comparison with a practically monophasic solid solution structure of Inconel 600 superalloy. Inconel 600 and 625 superalloys show minimal hardness in the HAZ of the joint and lower hardness of the seam area compared to the base material, while joints of Inconel 718 superalloys show a clearly higher hardness of the weld area and lower hardness of HAZ and parent material. The high hardness of Inconel 718 is connected with the presence of fine dendrites that solidify in the solid-liquid crystal zones at the fusion line of the weld, what can be seen in image taken by SUPRA 35 by ZEISS by the reflected electron method at an accelerating voltage of 20 kV with a magnification of up to 10,000× (Figure 7a). With increase of welding energy, the hardness of weld and HAZ increase. It can be connected with the laves phase, (FeNiCr)_2_(NbMoTiSi), dissolving, that is, more efficiently in high temperatures, above 70% of melting temperature [44]. The EDAX EDS microanalyzer was used for the microanalysis of the chemical composition of single precipitates revealed on the metallographic samples (Figure 7b).

### 3.2. Temperature Measurement Results

**Figure 8:**
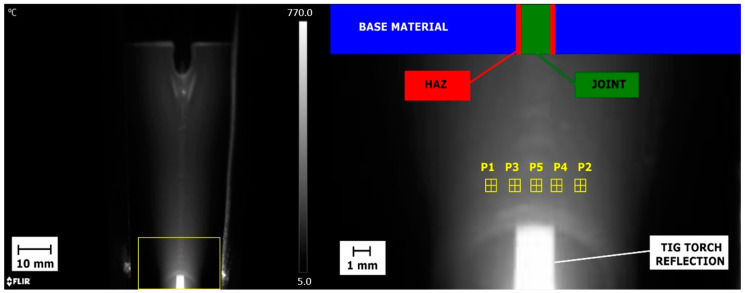
IR image taken during welding process. The welding torch is in the bottom of the images. Neighborhoods P1—P5 were used for assessment of temperature distribution in the seam area (P5), and the HAZ (P3, P4) and base material (P1, P2).

The IR measurement was made in such a way that in each thermogram, the welding torch, welding pool and solidified joint are visible (Figure 8). As the HAZ width is different for samples joined with various current setting. It varies from 0.2 to 0.6 mm, while the weld face has 1.8 to 4.9 mm. 

Selected ROIs, being the neighborhood of 3 × 3 pixels are used to measure temperature is HAZ and seam zones. This approach is used to minimize fluctuations of temperature caused by short hot spot appearance, that are a result of reflections caused by troch and welding table vibrations and by changes of joint face roughness. 

**Figure 9:**
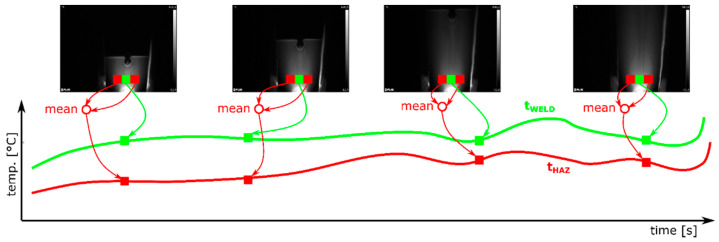
Idea of temporary temperature signal calculation form sequence of IR images to plots of temperature in joint area (T_WELD_) and in HAZ (T_HAZ_).

Areas P3 and P4 (Figure 8) are used to calculate temperature changes signal in the HAZ zone (Figure 9). For each IR image in the sequence, a mean value from P3 and P4 is calculated, to obtain T_HAZ_i_ temperature for the *i*th time moment. Temperature in the joint areas is taken directly dorm the P1 area, and it is for certain time moment the T_WELD_i_. For both signals, T_HAZ_ and T_WELD_ simple statistics, namely the mean and the median, and the standard deviation of the signal are calculated

**Figure 10:**
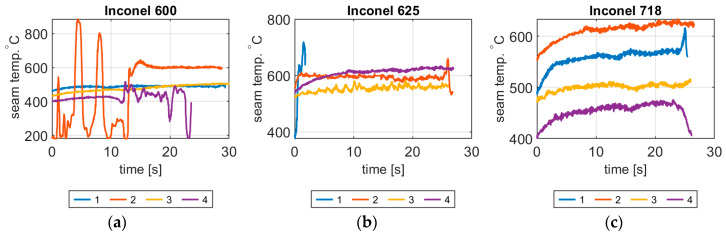
Temperature change during welding in the seam area (T_WELD_) for: (**a**) Inconel 600, A1–A4 samples, (**b**) Inconel 625, B1–B4 samples, (**c**) Inconel; 718, C1–C4 samples.

In Figure 10, temperature temporary plots generated for the seam area are shown. It can be seen, that increase of welding current (and linear energy) causes the increase of temperature generated on the joint surface. For the case A4, there are high temperature fluctuations. This is caused by the lack of fusion in this sample that is the result of low liner energy. In this case in the middle of seam disturbances caused by nozzles used to cover the ridge, because the thickness of weld layer is insufficient to exclude the influence of cold gas and hot nozzle housing on the measure results. Contrarily, for the A2 sample, variation of temperature is connected with the burnout. Statistical features of temperature signals extracted as explained earlier are gathered in Table 4.

### 3.3. Relationships between Parameters and Properties of Joints

To quantify the dependence between the temperature and mainly the hardness in joint and HAZ, the correlation between those parameters was calculated (Table 5). It can be seen, that for the Inconel 600 and 625 superalloys, the correlation between temperature (average, as well as median value) and hardness is high, and achieve more than *R* > 0.98. In the case there is negative correlation, because with increase of temperature (and linear energy) the hardness is decreasing. For Inconel 625 and 718, increasing the temperature, hardens is also increases. For HAZ in the case of Inconel 718, the correlation coefficient is lower as for other superalloy grades (*R* = 0.669 for avg. temperature and *R* = 0.736 for median temperature value).

**Figure 11:**
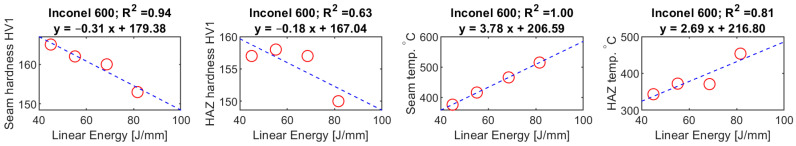
Regression model for Inconel 600 plates describing dependence between hardness in HAZ/joint areas and linear energy in those areas.

**Figure 12:**
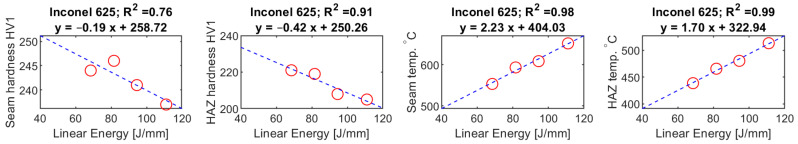
Regression model for Inconel 625 plates describing dependence between hardness in HAZ/joint areas and linear energy in those areas.

**Figure 13:**
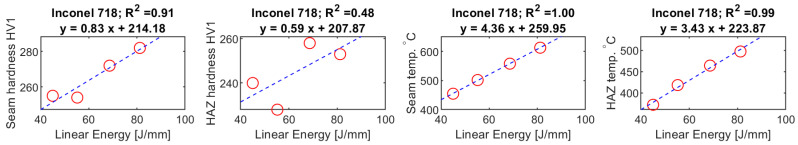
Regression model for Inconel 718 plates describing dependence between hardness in HAZ/joint areas and linear energy in those areas.

**Figure 14:**
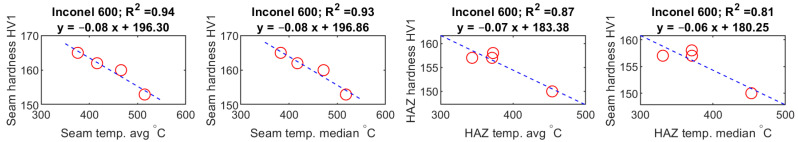
Regression model for Inconel 600 plates describing dependence between temperature in HAZ/joint areas and hardness in those areas.

The high dependence between temperature, linear energy and hardness, can be used to predict the mechanical properties of joint using IR camera. For prediction a model is needed to transform measured temperature to e.g., hardness. The simplest model can be obtained with means of linear regression. In the first step, a regression model was made to bound linear energy and hardness in HAZ and joint areas (Figure 11, Figure 12, Figure 13 and Figure 14). Linear energy was selected as the parameter describing the welding process, because there is no large variance of this value in time. This type of stable parameter can be also calculated taking int consideration only single, averaged values that can be read during the welding process. For Inconel 600 and 718 the regression fits the data quite well. The coefficient of determination, *R*^2^ = 1, was obtained for the liner energy and seam temperature for the 718 alloy. According to that proposed, linear model fits this type of parameters well. For Inconel 625, the correlation is at a lower level. To model Inconel 625 grade superalloys, application of the linear model is not an optimal solution. To reveal what was the problem and to determine the relationship between all signals, another model type should be used. 

**Figure 15:**
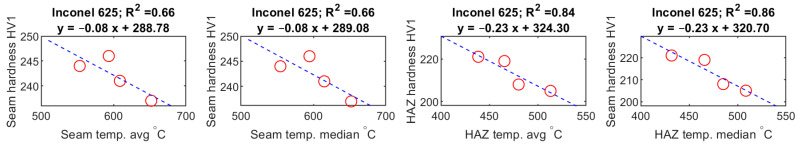
Regression model for Inconel 625 plates describing dependence between temperature in HAZ/joint areas and hardness in those areas.

**Figure 16:**
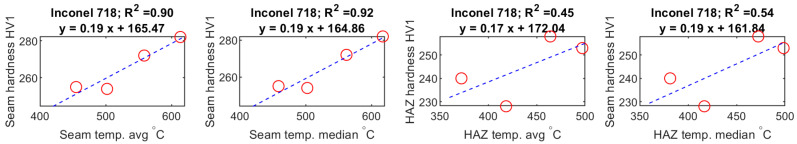
Regression model for Inconel 718 plates describing dependence between temperature in HAZ/joint areas and hardness in those areas.

**Figure 17:**
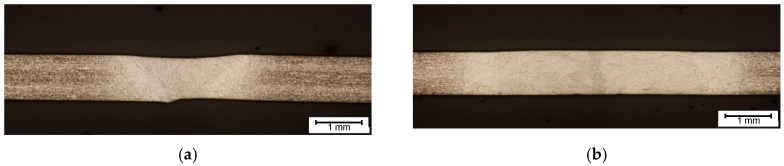

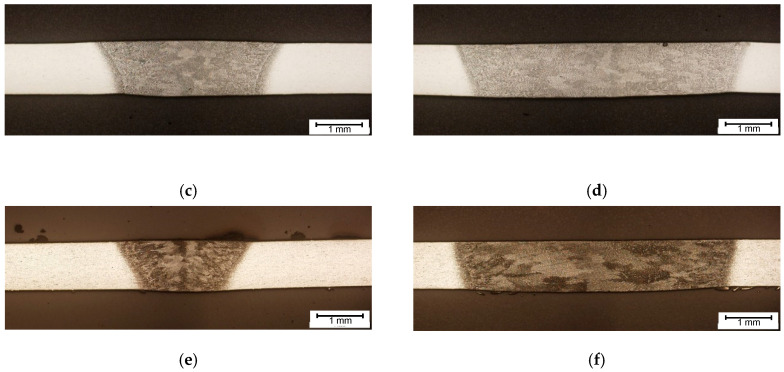
Weld cross sections for Inconel 600, 625 and 718 superalloys joints: (**a**) sample A4, E = 45 J/mm, (**b**) sample A2, E = 81.6 J/mm, (**c**) sample B3, E = 68.6 J/mm, (**d**) sample B4, E = 94.4 J/mm, (**e**) sample C4, E = 45 J/mm, (**f**) sample C2, E = 81.2 J/mm.

In practical, real-world, application of regression model it is favorable to assess the hardens in the joint. In this type of calculation correlation coefficients between consecutive variables (temp.—hardness) were obtained. The regressive model was trained separately for each seam localization handler. Obtained models are presented in Figure 14, Figure 15 and Figure 16. It can be seen, that for Inconel 600 and 718 based joints, the *R*^2^ ≥ 0.9. For the HAZ, the quality of model fit is lower, for Inconel 600 and 625 it does not exceed 0.87, while for Inconel 718, *R*^2^ = 0.54 for the pair of HAZ hardness and median temperature. The worse accuracy is caused mainly by the different seam and HAZ width for each specimen. While the distribution of ROIs used for temperature assessment is fixed, there is a risk, that the measurement that should be made for HAZ is made for joint or for base material zone. The joint width in set of specimens produced from one material type can change on about 200% between the minimal and the maximal one (Figure 17). Additionally, the linear model may be too much of a simplification compared to the actual relationship between temperature and hardness. Nevertheless, the result show, that there is a straightforward relationship between heat delivered to the joint and hardness obtained in joint and in HAZ.

To validate the obtained models, an additional set of joints was made for thin (1 mm) metal sheets made form Inconel 600 superalloy. Parameters of those joints are gathered in the Table 6. It can be seen, that measured values, as well as predicted ones keep the relationship, that for higher linear energy, the hardness decrease. Comparing measured and predicted values, the mean prediction error is as low as err = 1.25%. It can be stated that even such simple model, can be successfully applied to predict hardness in the joint (seam) area. 

Using linear model for predicting hardness in HAZ area, gave worse results, and the mean prediction error exceed err > 15%, what was highly unsatisfactory. This result relates to temperature assessment difficulties in the HAZ, because the joint face width for this material has the largest span (2.3 to 4.9 mm for Inconel 600) respecting the process parameters (Figure 17). Thus, having a constant positioned ROIs to measure temperature can lead to uncertain values. Nevertheless, it can be seen that full penetration for Ni superalloys thin sheets can be obtained taken various process parameters, leading to different geometrical and mechanical properties of joints.

## 4. Conclusions

In this paper, a study concerned on prediction of hardness in certain sections of welded joint for Inconel nickel superalloys. Assessing the temperature that was measured using infrared camera, a relation between obtained temperature values, welding linear energy and hardness that was obtained as the result of welding process in joint and HAZ. 

An increase in the linear energy of welding in the applied range causes an increase in the width of the weld and HAZ of the macroscopically examined joints. 

The maximum width of the HAZ as well as the face and root of the weld is demonstrated by the welded joints of Inconel 600 and 718 superalloys, with the highest linear energy of the arc being approximately 82 J/mm. Increasing the linear energy of the arc in the welding process of Inconel 600 and 718 superalloy joints in the range of 45 ÷ 81.6 J/mm causes in the HAZ an increase in the size of the γ matrix grains, respectively, from approximately 120 μm to approximately 200 μm and from approximately 10 μm to approximately 20 μm. 

In the HAZ in Inconel 625 superalloy samples, grain γ of approximately 40 μm was obtained, regardless of the welding energy used. The hardness in the tested zones of welded joints of Inconel 600, 625 superalloys and 718 depend significantly on their chemical composition but show a clear dependence on the welding test parameters used.

Inconel 600 and 625 superalloys show minimal hardness in the HAZ of the joint and lower hardness of the weld compared to that of the parent material, while joints of Inconel 718 superalloys show a clearly higher hardness of the weld area and lower hardness of HAZ and parent material.

A regressive model was elaborated and used to predict hardness values in joints. The mean prediction error for hardness of joint area at the level of err = 1.25% was obtained. According to that the proposed scheme can be applied for hardness prediction. Nevertheless, for the HAZ area, a further update of model type and measurement procedure is demanded to obtain predication accuracy on a similar level as for the seam (joint) region.

Further studies will cover update of prediction model. More complex regression approaches like ARMA/ARIMA modelling are taken into consideration, as well as machine learning approach with use of deep learning methods, where the input to the model will be in the form of whole IR image, not single temperature values. In the case of the regressive model, new method for assessing the temperature in HAZ will be proposed to overcome existing drawbacks connected with the variable width of seam and HAZ for different process parameters. There are 64 more test joints made for all three considered nickel superalloy grades for which thermogram sequences are acquires. For those samples, hardness will be measured, taking several cross sections of each made joint. Having a larger base of relations of temperature and hardness, a more accurate model will be elaborated.

## Figures and Tables

**Table 1:** Chemical composition of the investigated Inconel superalloys.

Super-Alloy	Element Concentration, wt %													
	Ni	Cr	Fe	Mo	Nb	Co	Mn	Cu	Al	Ti	Si	C	S	P
Inconel 600 *	74.43	15.76	8.60	-	0.08	0.05	0.25	0.09	0.18	0.27	0.12	0.01	0.002	0.005
Inconel 625 **	60.7	21.76	4.27	8.96	3.56	0.07	0.07	-	0.14	0.18	0.08	0.01	0.0003	0.007
Inconel 718 ***	52.24	18.19	19.61	2.89	5.13	0.13	0.06	0.01	0.60	1.00	0.08	0.05	0.0002	0.008

* Nb+Ta—0.08%; Ni+Co—74.48%, Ta—0.0002%, ** Nb+Ta—3.56%, N—0.01%, *** B—0.002%.

**Table 2:** Parameters of made joints.

Superalloy	ID	Welding Current I (A)	Average Arc Voltage U (V)	Linear Energy (J/mm)
Inconel 600	A1	35	9.8	68.58
	A2	40	10.2	81.6
	A3	30	9.2	55.2
	A4	25	9.0	45
Inconel 625	B1	50	11.1	111
	B2	40	10.2	81.6
	B3	35	9.8	68.58
	B4	45	10.5	94.5
Inconel 718	C1	35	9.8	68.58
	C2	40	10.2	81.6
	C3	30	9.2	55.2
	C4	25	9.0	45

**Table 3:** Hardness of specific zones measured for all welded specimens.

Superalloy	Linear Energy E J/mm	ID	Hardness HV1				
			Measurement Zone				
			BM/HAZ	HAZ	HAZ/S	S	BM
Inconel 600	68.6	A1	191	157	159	160	186
	81.6	A2	180	150	156	153	
	55.2	A3	193	158	162	162	
	45	A4	188	157	154	165	
Inconel 625	111	B1	248	205	234	237	253
	81.6	B2	225	219	245	246	
	68.5	B3	234	221	242	244	
	94.5	B4	244	208	237	241	
Inconel 718	68.6	C1	259	258	281	272	256
	81.2	C2	258	253	293	282	
	55.2	C3	239	228	281	254	
	45	C4	243	240	282	255	

**Table 4:** Temperature measured for the HAZ (T_HAZ_) and welded joint area (T_WELD_).

Superalloy	ID	Temperature °C					
		T_HAZ_			T_WELD_		
		Average	Standard Deviation	Median	Average	Standard Deviation	Median
Inconel 600	A1	370.95	5.40	371.16	465.75	19.72	471.50
	A2	453.81	3.73	453.04	514.67	16.41	517.88
	A3	372.30	15.55	370.92	416.01	7.51	417.27
	A4	343.48	23.49	331.07	376.00	18.96	382.68
Inconel 625	B1	513.25	12.86	508.57	652.03	11.23	652.13
	B2	465.61	6.89	465.84	593.41	12.02	594.50
	B3	438.61	14.97	431.87	553.22	10.76	554.39
	B4	480.29	10.24	485.21	608.72	19.69	614.96
Inconel 718	C1	464.12	23.87	472.41	558.37	17.61	561.91
	C2	497.14	10.75	498.52	613.35	18.19	617.09
	C3	418.44	10.67	416.73	501.94	10.15	502.38
	C4	372.32	21.71	381.40	454.90	14.67	458.71

**Table 5:** Correlation between temperature average and median (in HAZ and seam zones) and hardness/linear energy.

Superalloy	Correlation Argument	Correlation Factor			
		T_HAZ_		T_WELD_	
		Average	Median	Average	Median
Inconel 600	Hardness	−0.934	−0.899	−0.967	−0.963
	Linear Energy	0.899	0.924	1.000	1.000
Inconel 625	Hardness	−0.918	−0.929	−0.901	−0.921
	Linear Energy	0.994	0.988	0.989	0.993
Inconel 718	Hardness	0.669	0.736	0.950	0.957
	Linear Energy	0.994	0.992	1.000	1.000

**Table 6:** Results of hardness prediction in the seam area for Inconel 600 welded joints.

Sample ID	Welding Current [A]	Welding Speed [mm/s]	Linear Energy J/mm	Average Seam Temperature [°C]	Measured Hardness HV1	Predicted Hardness HV1	Prediction Error (err)
53	50	4	72.13	446.72	158	160	1.3%
54	50	4	72.21	451.78	157	160	1.9%
57	50	7	51.48	438.63	162	161	0.6%
58	50	7	51.49	438.00	163	161	1.2%

## Data Availability

The data presented in this study are available on request from the corresponding author. The data are not publicly available because the authors do not wish to publish supplementary materials.
